# Comment on Rivas et al. Unexpectedly Low Rate of Metastasis and Death Among Patients Treated for Uveal Melanoma with Brachytherapy, Vitrectomy, and Silicone Oil. *Cancers* 2025, *17*, 2683

**DOI:** 10.3390/cancers18081208

**Published:** 2026-04-10

**Authors:** Zelia M. Correa, J. William Harbour, Brian P. Marr, Prithvi Mruthyunjaya, David Reichstein, Miguel A. Materin, Amy C. Schefler, Alison Skalet

**Affiliations:** 1 Bascom Palmer Eye Institute, University of Miami Miller School of Medicine, Miami, FL 33136, USA; zcorrea@med.miami.edu; 2 Department of Ophthalmology, University of Texas Southwestern Medical Center, Dallas, TX 75390, USA; 3 Department of Ophthalmology, Columbia University, New York, NY 10032, USA; bpm2133@cumc.columbia.edu; 4 Byers Eye Institute, Stanford University, Stanford, CA 94305, USA; prithvi9@stanford.edu; 5 Tennessee Retina, Nashville, TN 37203, USA; david.reichstein@gmail.com; 6 Duke University Eye Center, Duke University, Durham, NC 27710, USA; 7 Retina Consultants of Texas, Houston, TX 77046, USA; acsmd@retinaconsultantstexas.com; 8 Casey Eye Institute, Oregon Health & Science University, Portland, OR 97239, USA; skalet@ohsu.edu; 9 Knight Cancer Institute, Oregon Health & Science University, Portland, OR 97239, USA

As the executive leadership of the Collaborative Ocular Oncology Group (COOG), which has conducted the largest prospective clinical studies ever performed in patients with uveal melanoma, we read with concern the recent report by Rivas, Samlowski and McCannel, which claims that brachytherapy combined with vitrectomy and silicone oil injection results in an “unexpectedly low rate” of metastasis and death in patients with uveal melanoma [1]. No clinical study to date has demonstrated a survival benefit of one treatment over another for uveal melanoma, provided that local control is achieved without recurrence [2]. Unfortunately, their study design is inadequate to contradict decades of scientific research and peer-reviewed literature.

Rivas et al. present a retrospective, uncontrolled study that included only 27 patients treated with brachytherapy, vitrectomy and silicone oil injection with a median follow-up of four years. Uveal melanoma is well known for its tendency to metastasize years after initial treatment and thus to require large cohorts and long follow-up to make statistically meaningful conclusions about survival. As such, a much larger number of study patients and propensity-matched control patients with much longer follow-up would be required to conduct a statistically credible study.

Despite the authors’ claim of improved survival outcomes with vitrectomy and silicone oil injection compared to historical data, the reported outcomes are virtually indistinguishable from those reported in the COOG Study Number 2 (COOG2), a recent large prospective multicenter study of 1577 uveal melanoma patients [3]. In this study, only 17.1% of patients developed metastasis after a median follow-up of 43.6 months, and the 5-year metastasis-free survival was estimated to be 79.9%. Importantly, none of the patients included in the COOG2 study underwent vitrectomy with silicone oil injection at the time of plaque brachytherapy.

Based on the overwhelming consensus of the scientific literature, metastatic risk in uveal melanoma is determined almost entirely by a small set of recurrent molecular alterations along with a small contribution from tumor size [3]. When these features are taken into consideration, the choice of primary treatment makes no contribution to metastatic risk or survival [3]. Contrary to the authors’ stated concern regarding “the potential inaccuracy of the ability of current prognostic testing to identify patients who will eventually metastasize,” the accuracy of molecular prognostic testing by gene expression profiling (GEP) has been shown by numerous studies to be very high in this cancer. Indeed, the COOG has prospectively validated the prognostic accuracy of GEP in two multicenter studies totaling over 2000 patients, providing the highest level of biomarker evidence in this cancer type and demonstrating superiority over monosomy 3 and anatomic staging systems [3,4].

Another concern with this study is the lack of biological rationale for how vitrectomy or silicone oil injection could alter metastatic risk. The authors suggest that removing the vitreous may reduce tumor burden, but this is illogical since enucleation accomplishes an even more complete removal of the vitreous and tumor burden, yet enucleation has clearly been shown to offer no survival benefit over other treatment modalities [2]. Further, we can find no evidence in the scientific literature that silicone oil, which is considered to be biologically inert, may have a tumoricidal effect on cancer cells.

Given these serious concerns, we remain highly skeptical that vitrectomy and silicone oil injection improve visual outcomes, much less that these techniques improve survival in patients with uveal melanoma. Vitrectomy and silicone oil injection at the time of brachytherapy is not standard-of-care and is not widely performed, given the lack of evidence-based benefit—ocular or systemic. In the absence of validation in larger, prospective, well-powered and properly controlled studies, any claims of survival benefit from vitrectomy with silicone oil are highly speculative and potentially misleading.

## References

[1] Rivas A., Samlowski W., McCannel T.A. (2025). Unexpectedly Low Rate of Metastasis and Death Among Patients Treated for Uveal Melanoma with Brachytherapy, Vitrectomy, and Silicone Oil. Cancers.

[2] Diener-West M., Earle J.D., Fine S.L., Hawkins B.S., Moy C.S., Reynolds S.M., Schachat A.P., Straatsma B.R., Collaborative Ocular Melanoma Study Group (2001). The COMS randomized trial of iodine 125 brachytherapy for choroidal melanoma, III: Initial mortality findings. COMS Report No. 18. Arch. Ophthalmol..

[3] Harbour J.W., Correa Z.M., Schefler A.C., Mruthyunjaya P., Materin M.A., Aaberg T.A., Skalet A.H., Reichstein D.A., Weis E., Kim I.K. (2024). 15-Gene Expression Profile and PRAME as Integrated Prognostic Test for Uveal Melanoma: First Report of Collaborative Ocular Oncology Group Study No. 2 (COOG2.1). J. Clin. Oncol..

[4] Onken M.D., Worley L.A., Char D.H., Augsburger J.J., Correa Z.M., Nudleman E., Aaberg T.M., Altaweel M.M., Bardenstein D.S., Finger P.T. (2012). Collaborative Ocular Oncology Group report number 1: Prospective validation of a multi-gene prognostic assay in uveal melanoma. Ophthalmology.

